# Author Correction: Probabilistic Assessment of Nerve Regeneration with Diffusion MRI in Rat Models of Peripheral Nerve Trauma

**DOI:** 10.1038/s41598-020-64339-z

**Published:** 2020-04-29

**Authors:** Isaac V. Manzanera Esteve, Angel F. Farinas, Alonda C. Pollins, Marlieke E. Nussenbaum, Nancy L. Cardwell, Hakmook Kang, Mark D. Does, Wesley P. Thayer, Richard D. Dortch

**Affiliations:** 1 Vanderbilt University Medical Center, Department Radiology and Radiological Sciences, Nashville, TN USA; 2 Vanderbilt University Medical Center, Institute of Imaging Science, Nashville, TN USA; 3 Vanderbilt University Medical Center, Department of Plastic Surgery, Nashville, TN USA; 4 Vanderbilt University Medical Center, Department of Biostatistics, Nashville, TN USA; 5 Vanderbilt University, Department of Biomedical Engineering, Nashville, TN USA

Correction to: *Scientific Reports* 10.1038/s41598-019-56215-2, published online 23 December 2019

This Article contains errors in the Methods sections under subheading ‘MRI protocol’.

“Images were acquired with a three-dimensional diffusion-weighted spin-echo sequence and the following parameters: field-of-view = 60 × 60 × 160 mm^3^, resolution = 125 × 125 × 372 µm^3^, TE/TR = 22/425 ms, gradient pulse duration/diffusion time (δ/Δ) = 4/12 ms, b-values = 2000 and 4000 s/mm^2^, 20 diffusion directions, number of averaged excitations = 2, and scan time = 7 hours and 40 minutes for each b-value.”

should read:

“Images were acquired with a three-dimensional diffusion-weighted spin-echo sequence and the following parameters: field-of-view ≈ 6 × 6 × 16 mm^3^ (adjusted to fit sample), resolution = 125 × 125 × 372 µm^3^, TE/TR = 22/425 ms, gradient pulse duration/diffusion time (δ/Δ) = 4/12 ms, b-values = 2000 and 4000 s/mm^2^, 20 diffusion directions, number of averaged excitations = 2, and scan time ≈ 7-10 hours for each b-value (depending on the field-of-view).”

