# Supplementary material for: Probabilistic Assessment of Nerve Regeneration with Diffusion MRI in Rat Models of Peripheral Nerve Trauma
Source: Sci Rep. 2019 Dec 23;9:19686. doi: 10.1038/s41598-019-56215-2 (PMC6928159; doi:10.1038/s41598-019-56215-2)
Supplement: Supplementary file 1 — Supplementary Figure S1 [file 41598_2019_56215_MOESM1_ESM.pdf]

Supplementary Information for

Probabilistic Assessment of Nerve Regeneration with Diffusion MRI in Rat Models of Peripheral Nerve Trauma

Isaac V. Manzanera Esteve[1,2], Angel F. Farinas[3], Alonda C. Pollins[3], Marlieke E. Nussenbaum[3], Nancy L. Cardwell[3], Hakmook Kang[4], Mark D. Does[1,2,5], Wesley P. Thayer[3,5], and Richard D. Dortch[1,2,5,*]

[1] Vanderbilt University Medical Center, Department Radiology and Radiological Sciences, Nashville, TN

[2] Vanderbilt University Medical Center, Institute of Imaging Science, Nashville, TN

[3] Vanderbilt University Medical Center, Department of Plastic Surgery, Nashville, TN

[4] Vanderbilt University Medical Center, Department of Biostatistics, Nashville, TN

[5] Vanderbilt University, Department of Biomedical Engineering, Nashville, TN

Corresponding author:

Richard D. Dortch

Department of Radiology and Radiological Sciences

Vanderbilt University Medical Center

1161 21st Avenue South

Medical Center North, AA-1105

Nashville, TN 37232-2310

Phone: 615-875-8792

Email: richard.dortch@vanderbilt.edu

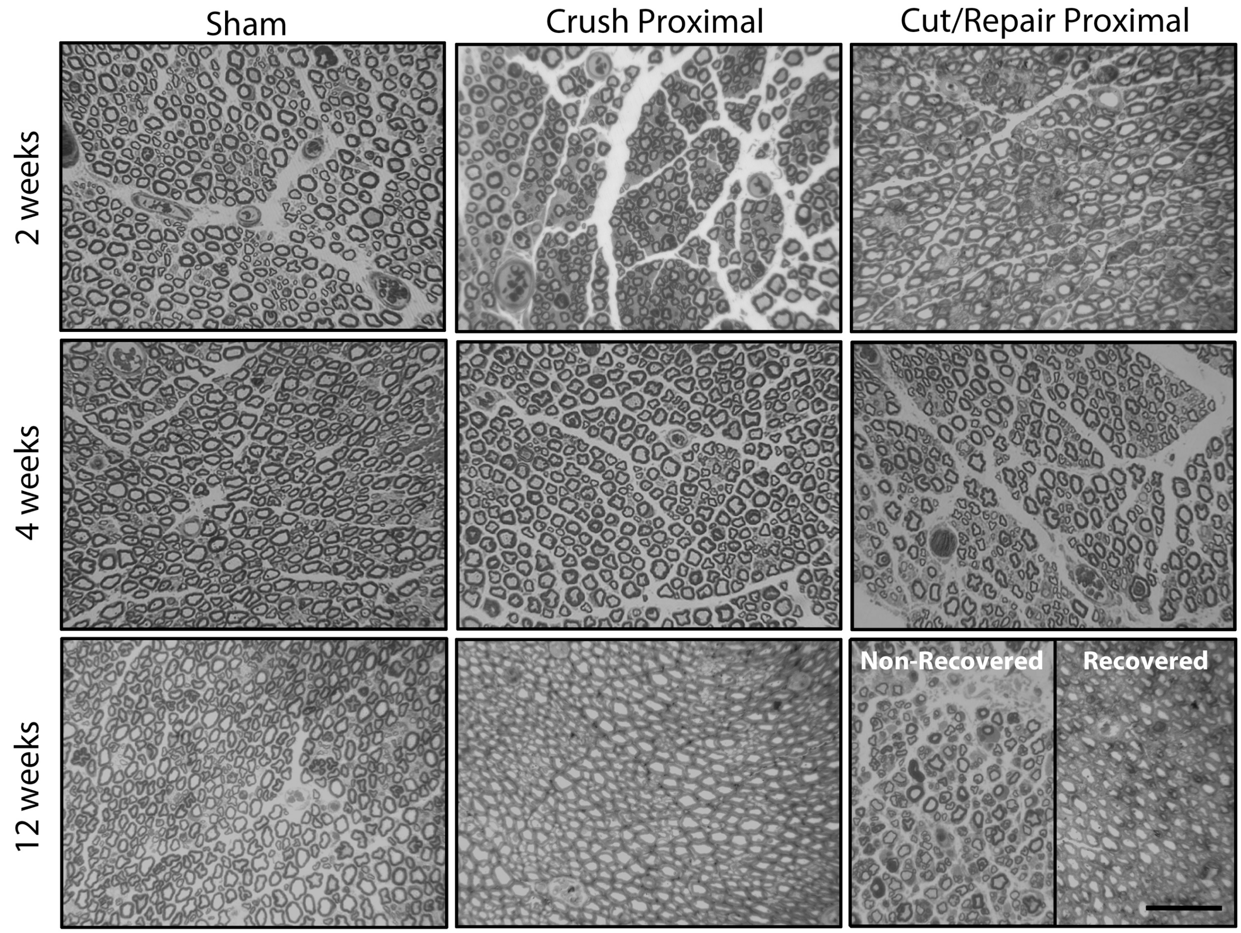

Figure S1: Representative proximal toluidine blue stained sections at 2 (top panel), 4 (middle panel) and 12 weeks (bottom panel) after each injury type. In the sham groups (left column), there was little difference between the three times, except for some evidence of extra-cellular edema at 2 weeks. Note the absence of nerve degeneration in the other cohorts, including cut/repair nerves that showed little ("non-recovered" panel) and partial recovery ("recovered" panel). Together with the distal histology in Figure 8, which showed evidence of nerve degeneration, these findings suggest that we are detecting axonal sprouting with our DTI measures. The scale bar in the bottom-right panel represents 50 μm.
